# A RALGEF inhibitor suppresses lung cancer stem cell development and tumorigenesis

**DOI:** 10.1016/j.jbc.2026.113564

**Published:** 2026-09-16

**Authors:** Raphael Jigo, Geoffrey J. Clark

**Affiliations:** Department of Pharmacology and Toxicology, University of Louisville, Louisville, Kentucky, USA

**Keywords:** RAS, RAL, RALGDS, lung cancer, cancer stem cells

## Abstract

Genetic studies have implicated RAL proteins as key components of RAS-mediated transformation and metastasis. They can modulate drug resistance, the cancer stem cell population and the exocyst transport system. RAL proteins are activated by RALGEFs (Guanine Nucleotide Exchange Factors). A conserved family of four RALGEFs serve as direct effectors of the RAS oncoprotein, connecting RAS to the regulation of RAL proteins. We have previously reported the development of a first-in-class small molecule inhibitor of RALGEFs that acts by binding to the RAS association domain of RALGEFs, blocking their interaction with RAS and suppressing the activation of RAL. Here we show the agent is active in KRAS-driven non-small cell lung cancer (NSCLC) models, both *in vitro* and *in vivo* using cell line and transgenic models of lung cancer. Moreover, we show that it acts to suppress the anti-porter activity of the RAL effector protein RALBP1, sensitises cells to standard-of-care treatment and suppresses the exocyst system. Finally, we show that it acts to suppress the cancer stem cell pool using biomarkers and tumor-forming unit assays. Thus, we identify a potential novel therapeutic approach to NSCLC.

Lung cancer remains the most lethal and frequently diagnosed cancer type worldwide, accounting for 12% of new cancer diagnoses and 19% of cancer-related deaths (1). Lung tumors are composed of two main subtypes: small cell (SCLC) and non-small cell lung cancer (NSCLC). NSCLC tumors are the most frequently diagnosed (85%) (2). Genomic studies have identified multiple oncogenic drivers of NSCLC (3, 4), with KRAS being the most frequently mutated (3, 5). Targeting KRAS, therefore, has long been an attractive therapeutic approach for this disease.

Active RAS exerts its biological effects by binding and activating downstream signaling effectors, the most characterized being the kinase Raf and the p110 catalytic subunit of PI3K, leading to activation of the MAPK and PI3K pathways, respectively (6). In addition, RAS can also bind to a less studied third class of effector proteins called RALGEFs (Ral Guanine Nucleotide Exchange Factors). Four RAS effector RALGEF family members have been identified, the best characterized being RALGDS (7, 8). Binding of RAS to RALGDS stimulates the cycling of GDP for GTP on downstream RALA and RALB proteins, promoting their activation (8, 9). Active RAL proteins then bind a multitude of downstream effector proteins to modulate cellular processes such as cell cycle progression, exo- and endocytosis, and actin dynamics, among others (10).

However, one of the most important functions of the RAL pathway may be its involvement in the modulation of cancer stem cells (CSC) (11, 12, 13) - a small, intratumoral subpopulation of cells with the capability of self-renewal, differentiation, and tumorigenicity. RALA is required for intestinal stem cell regeneration downstream of Wnt signaling, and its knockout reduces stem cell function while increasing sensitivity to Wnt inhibition (13). Furthermore, a KRAS/RALB/Sec5/TBK1/NF-B pathway has been identified (14) as necessary and sufficient for stem cell regeneration, tumor initiation, and anchorage-independence in EGFR-positive tumors (15). High RAL activation has also been observed in cancer stem cells derived from lung adenocarcinoma and hepatocellular carcinomas (11, 16). Due to their role in metastasis, chemotherapy resistance, and tumor relapse (17, 18), targeting this subpopulation of tumor initiating cells and its regulatory signaling pathways is an area of considerable scientific interest.

Examination of a panel of pancreatic cell lines showed that the RAL pathway exhibited a higher level of activation in comparison to activation of the MAPK and PI3K effector pathways (19). Lung adenocarcinoma cell lines have also been demonstrated to exhibit elevated RAL pathway activation (11). Moreover, in multiple cancer types, suppression or knockout of RALA or RALGDS suppresses tumor development and metastasis, although exerting limited effects on *in vitro* 2D cellular proliferation (11, 16, 19, 20, 21, 22). Thus, the RAS/RALGEF signaling pathway appears to be essential for RAS-driven cancer development (19, 23). To date, no FDA-approved inhibitors of this pathway exist.

Targeting RAS directly has historically proved very challenging, and for many years the protein was considered “undruggable”. However, the approval of Sotorasib in 2021 and Adagrasib soon after, both of which specifically target the G12C mutant of KRAS, demonstrated targeting RAS directly as a feasible approach against NSCLC tumors (24). Although a significant milestone, non-G12C KRAS-mutated NSCLC (∼60% of mutant RAS tumors) is not vulnerable to these drugs (25). Moreover, resistance to anti-G12C therapy is frequent and often quite rapid (26). Examination of the molecular effects of cells resistant to Sotorasib has shown an unexpected activating effect on AKT (27), and treatment with Sotorasib is ineffective against the RAS/RALGEF/RAL pathway (28). Whether these effects are due to feedback loops, off-target effects, or compensation from non-KRAS^G12C^ RAS isoforms is unclear.

An alternative approach to combating these cancers has been to target signaling downstream of RAS, with numerous approved inhibitors for the most characterized MAPK and PI3K effector pathways existing in the clinic today (29). Development of resistance to these inhibitors, however, is also observed after prolonged treatment, with attempts at combinatorial approaches to address this yielding high toxicity profiles (29).

We have recently described a first-in-class pan-RALGEF inhibitor which shows anti-tumor activity against KRAS-driven pancreatic cancer models (28). However, we had not defined the mechanism of action. Here, we show the agent is also active against RAS-driven *in vitro* and *in vivo* models of NSCLC. Moreover, we show it suppresses activation of the two main RAL effector systems, RALBP1 and the exocyst. In addition, we show the agent can suppress the CSC compartment in mutant KRAS NSCLC cell systems while a KRAS-specific inhibitor does not. This highlights the potential of the RALGEF inhibitor not just as a single agent but also as a future combination therapy with the new RAS inhibitors entering the arena.

## Results

### C4-180 inhibits 3D but not 2D growth of mutant RAS NSCLC cells

We have previously described a pan-RALGEF inhibitor, designated C4-180, that suppresses RAL activation and is active against RAS-driven pancreatic cancer systems (28). The agent acts preferentially on 3D cell growth *in vitro*. This is consistent with genetic inhibition of this pathway, as knockdown of RALA (19) or the RALGEF RGL2 (30) is dispensable for pancreatic cancer cell proliferation but suppresses 3D growth and tumor formation.

To determine the effects of the compound on mutant RAS NSCLC, we performed 3D soft agar growth assays on two mutant RAS NSCLC cell lines. After 2 weeks of incubation, C4-180 significantly suppressed 3D tumor colony formation (Fig. 1*A*). To test whether any effects could be observed in 2D, we treated cells using a concentration shown to suppress about 50% of colonies in our 3D soft agar assays. No significant difference in 2D cell number was observed in treated *versus* non-treated cells (Fig. 1*B*). RAL activation assays confirmed suppression of RAL activity (Fig. 1*C*).

**Figure 1:**
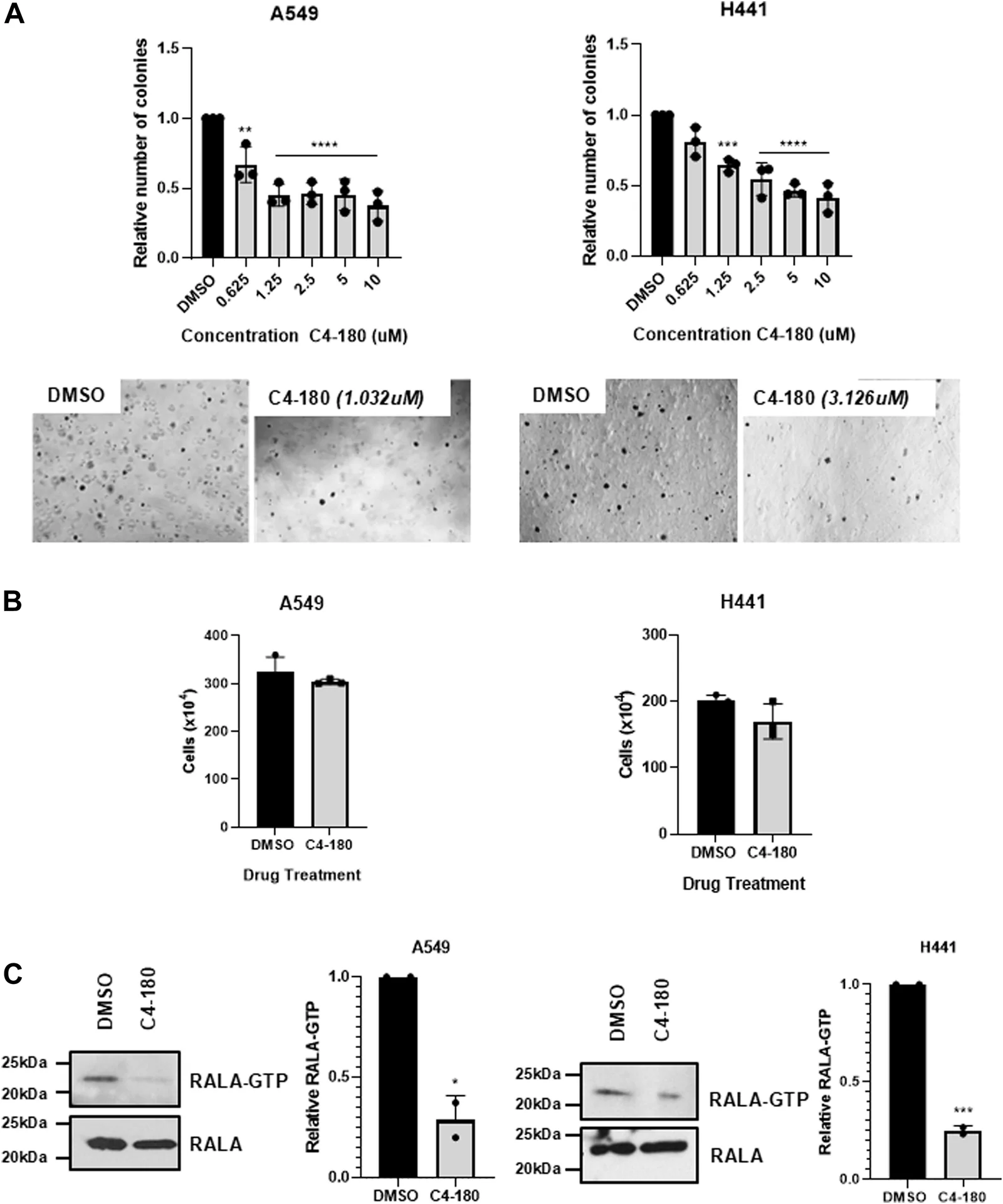
**3D vs 2D growth effects of C4-180 treatment.***A*, effects of treatment with C4-180 on 3D soft agar colony formation of A549 (K-RAS^G12S^) and H441 (KRAS^G12V^) lung adenocarcinoma cell lines. Colonies were scored in duplicate for each concentration after 2 weeks of incubation. Graphs are representative of growth relative to DMSO control. Significance was determined by one-way ANOVA followed by Dunnet’s multiple comparisons test (n = 3. ∗∗ = *p* < 0.01, ∗∗∗ = *p* < 0.001 ∗∗∗∗ = *p* < 0.0001). Images are representative of agar assays at concentrations shown to inhibit approximately 50% of colonies. *B*, effects of treatment on 2D cell growth over the same period of incubation as 3D soft agar assays at a dose of 2.5 μM, a concentration shown to suppress 3D colony formation. Significance was determined by *t* test (n = 3, no significance seen). *C*, A549 and H441 cells were transiently treated for 2 h with C4-180 (10 μM), followed by determination of active RALA-GTP levels using a commercial RAL Activation Assay Kit. Graphs represent RALA-GTP levels relative to total RALA protein levels. Significance determined by *t* test (n = 2, ∗ = *p* < 0.05, ∗∗∗ = *p* < *p* < 0.001). Error bars are SD.

### C4-180 impairs RALBP1 function and sensitizes cells to doxorubicin treatment

One of the major Ral effector proteins is RALBP1. RALBP1 serves as a multidrug transporter for several chemotherapeutic drugs and is often overexpressed during the development of drug resistance (31). It is has been identified as a transporter of and mediator of resistance to doxorubicin (32), with its knockout leading to increased intracellular doxorubicin accumulation, and enhanced cell death (31, 32). To investigate whether our agent was affecting RALBP1 activity, we tested whether C4-180 could suppress efflux of doxorubicin by first pre-treating cells with C4-180 for 2 h, followed by treatment with doxorubicin for another hour, after which the media were refreshed with drug-free media. Intracellular accumulation of doxorubicin was measured through quantification of red fluorescent area in both control and C4-180-treated conditions. Treatment with C4-180 caused a significant increase in intracellular retention of doxorubicin over a 24-h period when compared to DMSO, coinciding with a significant enhancement of the effects of doxorubicin on cell growth in comparison to treatment with doxorubicin alone (Fig. 2). These results demonstrated the activity of C4-180 against RalBP1 function in drug transport and chemoresistance.

**Figure 2:**
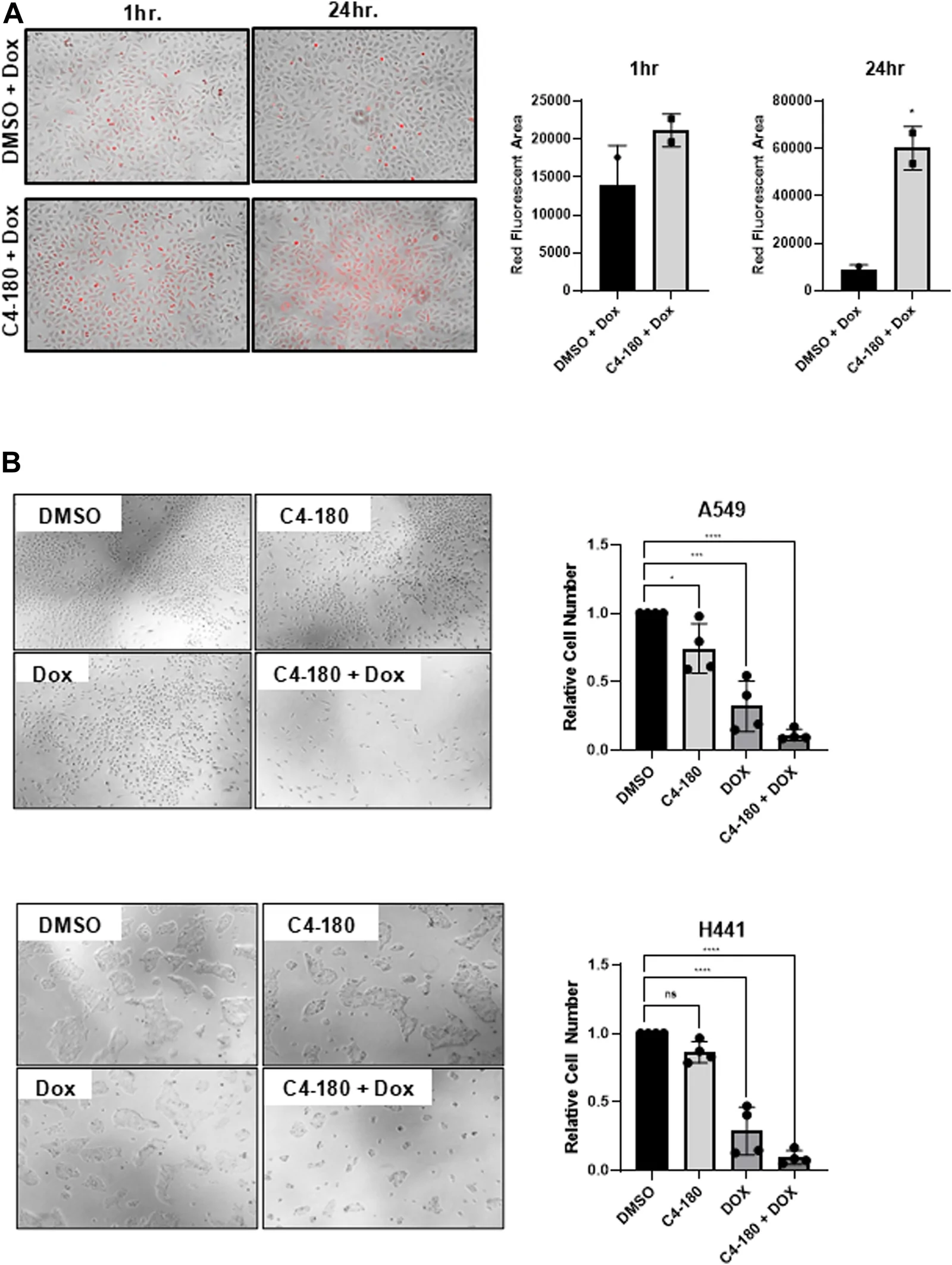
**C4-180 suppresses RALBP1 efflux transport of doxorubicin.***A*, effects of C4-180 on cellular doxorubicin retention. A549 cells were pretreated with C4-180 (10 μM) for 2 h, followed by treatment with doxorubicin (10 μM), after which cells were refreshed with drug-free media. *Red* fluorescence area was imaged at 1 h and 24 h time points. Quantification of *red*, fluorescent area was done using ImageJ. Students *t* test was used to determine significance (n = 2. ∗ = *p* < 0.05). (*B*) Effects of C4-180 (5 uM) and doxorubicin treatment (2 nM) over a 3-day period in A549 and H441 cells. Student *t* test was used to determine significance between the shown pairs (n = 4. ∗ = *p* < 0.05, ∗∗ = *p* < 0.01, ∗∗∗*p* < 0.001). Error bars are SD.

### The C4-180 RALGEF inhibitor impairs exocyst dynamics

The exocyst is an octameric protein complex involved in the delivery and tethering of intracellular vesicles to the plasma membrane (33). It has been shown to regulate the delivery of molecules such as EGFR and E-cadherin to the basolateral membrane in epithelial cells. RAL proteins can bind the Sec5 and Exo84 subunits of the exocyst complex to regulate its function (34, 35). The exocyst complex associates with RAB-positive recycling endosomes, regulating their function in transporting and tethering endosomal cargo back to the membrane (36). The Wnt receptor frizzled has been shown to be directed to Rab-positive recycling endosomes to promote its membrane transport (37). We thus investigated whether treatment with our anti-RALGEF agent could block exocyst dynamics by using Wnt receptor Fzd4 as a marker of RAL regulation of the exocyst. To test this, a GFP-Fzd4 construct was transfected into A549 cells and allowed to express for 24 h. Fzd4 normally maintains a primarily membrane-localized expression (Fig. 3). Treatment with C4-180 caused an increased perinuclear colocalization of GFP-Fzd4 with lysosomes and lesser membrane-bound expression of Fzd4 (Fig. 3), implicating modulation of exocyst dynamics by C4-180. As a control, we included a MEK inhibitor (Trametinib) which acts on the RAS/MAPK pathway but has no effect on the RAS/RAL pathway. It had no effect.

**Figure 3:**
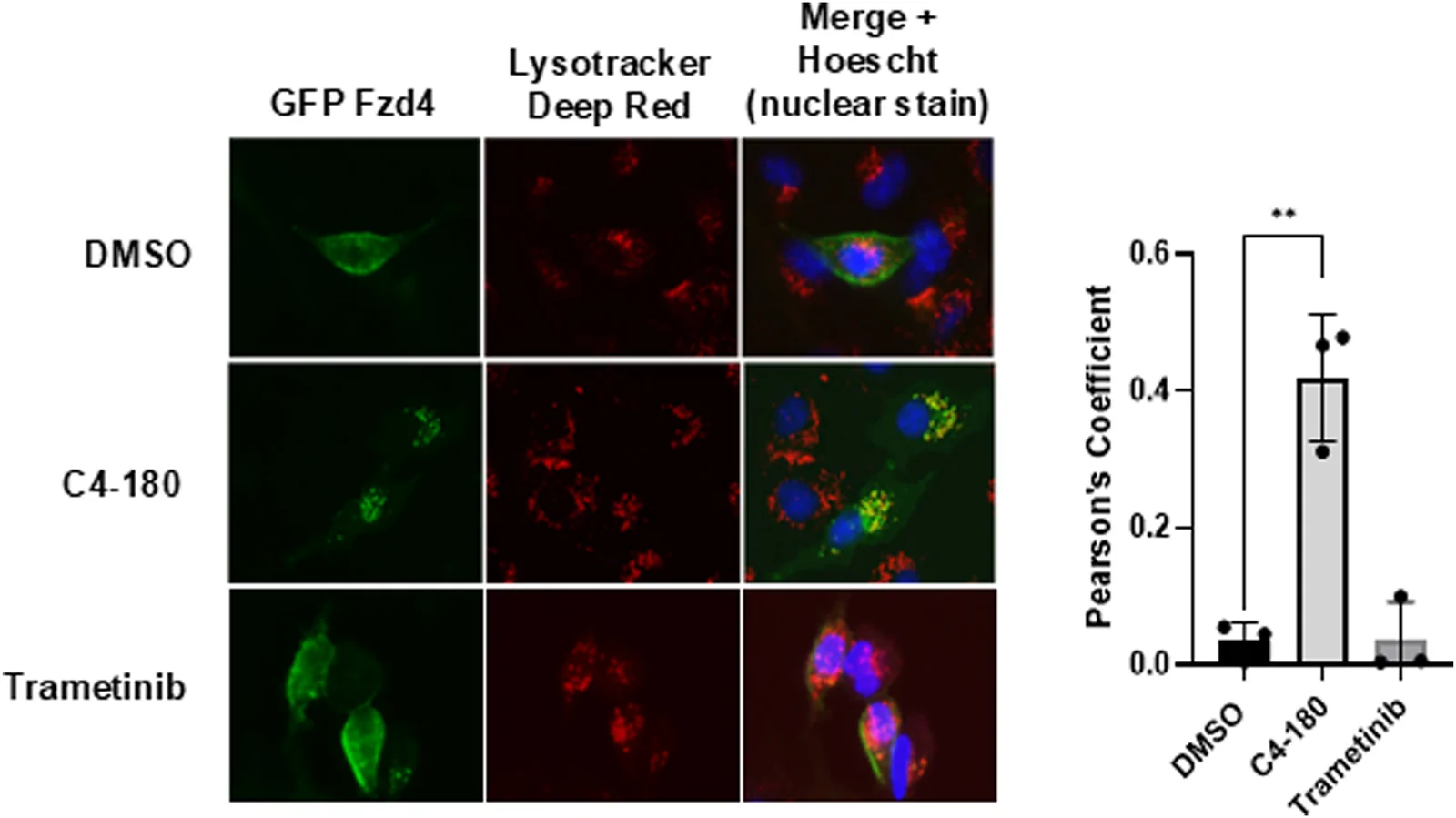
**C4-180 impairs exocyst dynamics.** GFP-Fzd4 plasmid was transfected into A549 cells, allowed to incubate for 24 h, followed by overnight treatment with DMSO, C4-180 (10 μM), or trametinib (100 nM). Following drug treatment, culture media were refreshed with 75 nM of LysoTracker Deep *Red* (Invitrogen, catalog no. L12492) reagent for 30 min to visualize perinuclear-localized lysosomes. Colocalization with GFP-Fzd4 was confirmed using JaCoP plugin extension for ImageJ to generate Pearson’s Coefficient value. Percentage of membrane vs. perinuclear localized Fzd4 was determined for each condition through manual scoring of 100 cells for each condition (n = 3. *t* test used to determine significance. ∗∗ = *p* < 0.01). Error bars are SD.

### Inhibition of RAL pathway activation and cancer stem cell (CSC) markers

Previous studies have shown the formation of colonies in 3D culture to be driven by the CSC subpopulation (38). The RAL pathway has been shown to be essential for stem cell maintenance and function. It is highly activated in CSCs which comprise only a small percentage of the intra-tumoral bulk population (11, 12, 13, 15, 16). Moreover, both RALBP1 (39) and the Wnt pathway (shown to be regulated by RAL) regulate stem cells (13). Therefore, we sought to determine if our pan-RALGEF inhibitor was mediating its biological effects, in part, by suppressing the CSC population. We first confirmed RAL pathway inhibition using a commercial RAL pulldown assay kit. RALA activation was significantly suppressed after treatment with C4-180 (Fig. 4*A*). Treatment with a commercial direct pan-KRAS inhibitor (BI-2865) suppressed phosphorylation of ERK, demonstrating high activity against the RAS/MAPK; however, it exhibited little effect on the RAS/RALGEF pathway (Fig. 4*A*).

**Figure 4:**
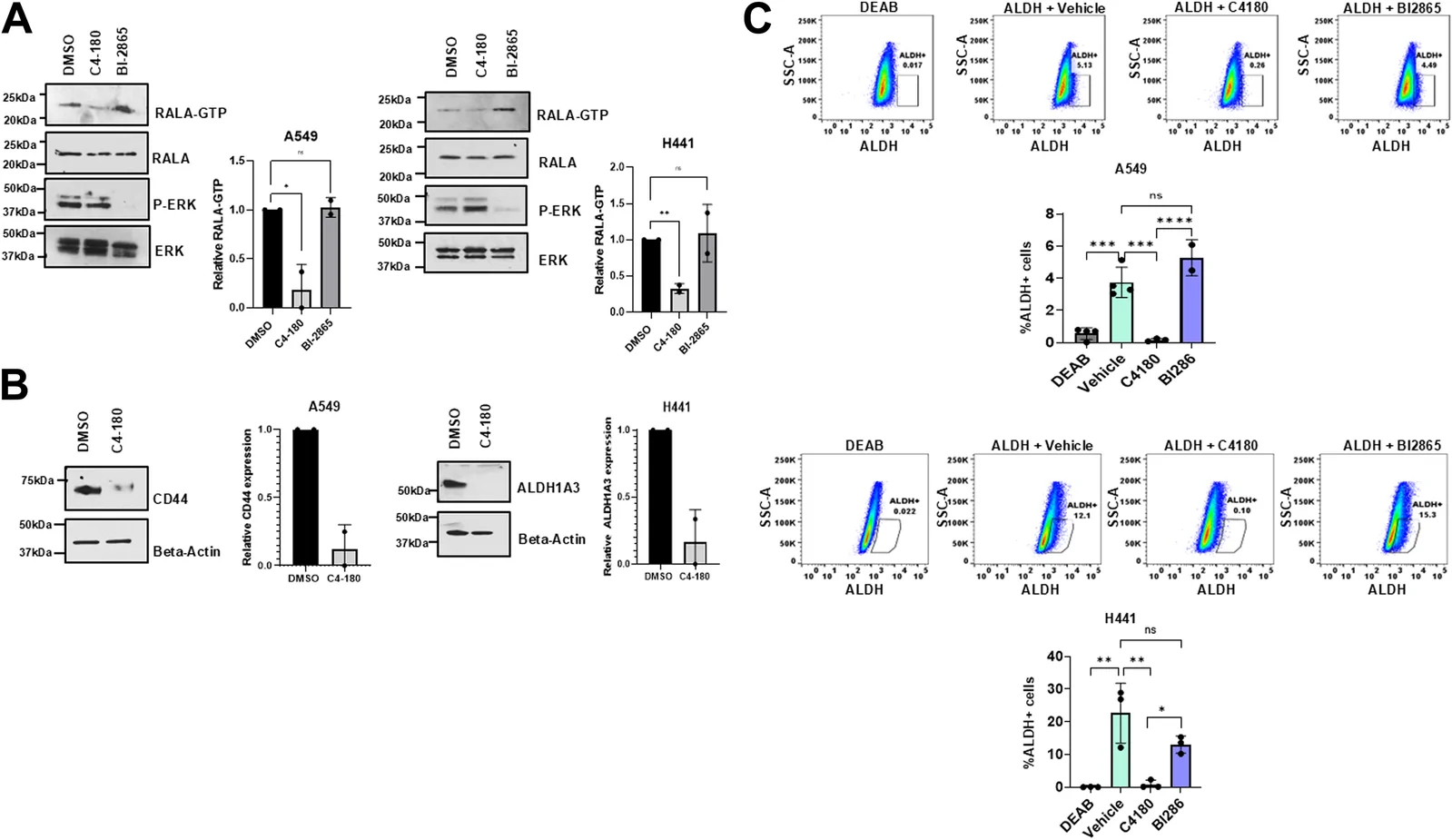
**C4-180 suppresses markers of stemness in lung adenocarcinoma cell lines.***A*, comparison of relative effects on RALA activation when comparing treatment with C4-180 (10uM) and pan K-RAS inhibitor BI-2865 (2.5uM). (n = 2; Student *t* test used to determine significance). *B*, effects of C4-180 (10 μM) on CSC stem cell markers CD44 and ALDH1A3 in A549 and H441 cells, respectively. (n = 2). *C*, A549 and H441 cells were treated with either DMSO, C4-180 (2.5uM), or BI-2865 (2.5 uM) for 7 days, before determination of effects on ALDH activity was carried out using an Aldefluor assay kit according to the manufacturer’s protocol. Representative histograms are gated based on the negative DEAB control to determine ALDH^high^ population. Bar graphs represent the percentage of ALDH^high^ positive cells in comparison to the total cell population. (n = 3; analyzed by *t* test between pairs ∗∗ = *p* < 0.01, ∗∗∗ = *p* < 0.001 ∗∗∗∗ = *p* < 0.0001). Error bars are SD.

CSCs are identified and isolated based on a combination of properties, including the expression or activity of extracellular and intracellular markers (*e.g.*, CD44, CD133, ALDH1, *etc.*) (17). These markers have been validated as identifiers of tumour-initiating cells due to their ability to form tumors *in vivo*, and cell line-specific CSC markers of NSCLC cell lines have previously been characterized (40). In drug treatment assays, C4-180 was sufficient to suppress protein expression of CSC markers CD44 and ALDH1A3 in A549 and H441 cells, respectively (Fig. 4*B*).

Elevated ALDH activity has been validated as a marker for and is observed in stem cell populations (41). To further confirm activity against the CSC subpopulation, a commercial Aldefluor kit was used to determine the percentage of ALDH^high^ cells in treated vs. non-treated control.

Treatment with C4-180 resulted in suppression of the ALDH^high^ population, almost comparable to DEAB (an inhibitor of ALDH1 enzymes), which serves as a negative control and establishes gating parameters for this assay (Fig. 4*C*). Surprisingly, treatment with BI-2865 at the same concentration showed no suppressive effect on this population (Fig. 4*C*).

The definitive assessment of CSC function is the ability to form tumors in mice (42). As we observed suppression of CSC markers in our treatment assays, we sought to determine whether this treatment would also coincide with a decreased capability for tumor formation *in vivo*. When cells were pre-treated at the same concentration and length of time, followed by subcutaneous implantation of viable cells in NSG mice and no further treatment, we observed almost complete loss of tumor-forming capability from the cell populations (Fig. 5). MTT assays were used to confirm equal input cell viability prior to implantation.

**Figure 5:**
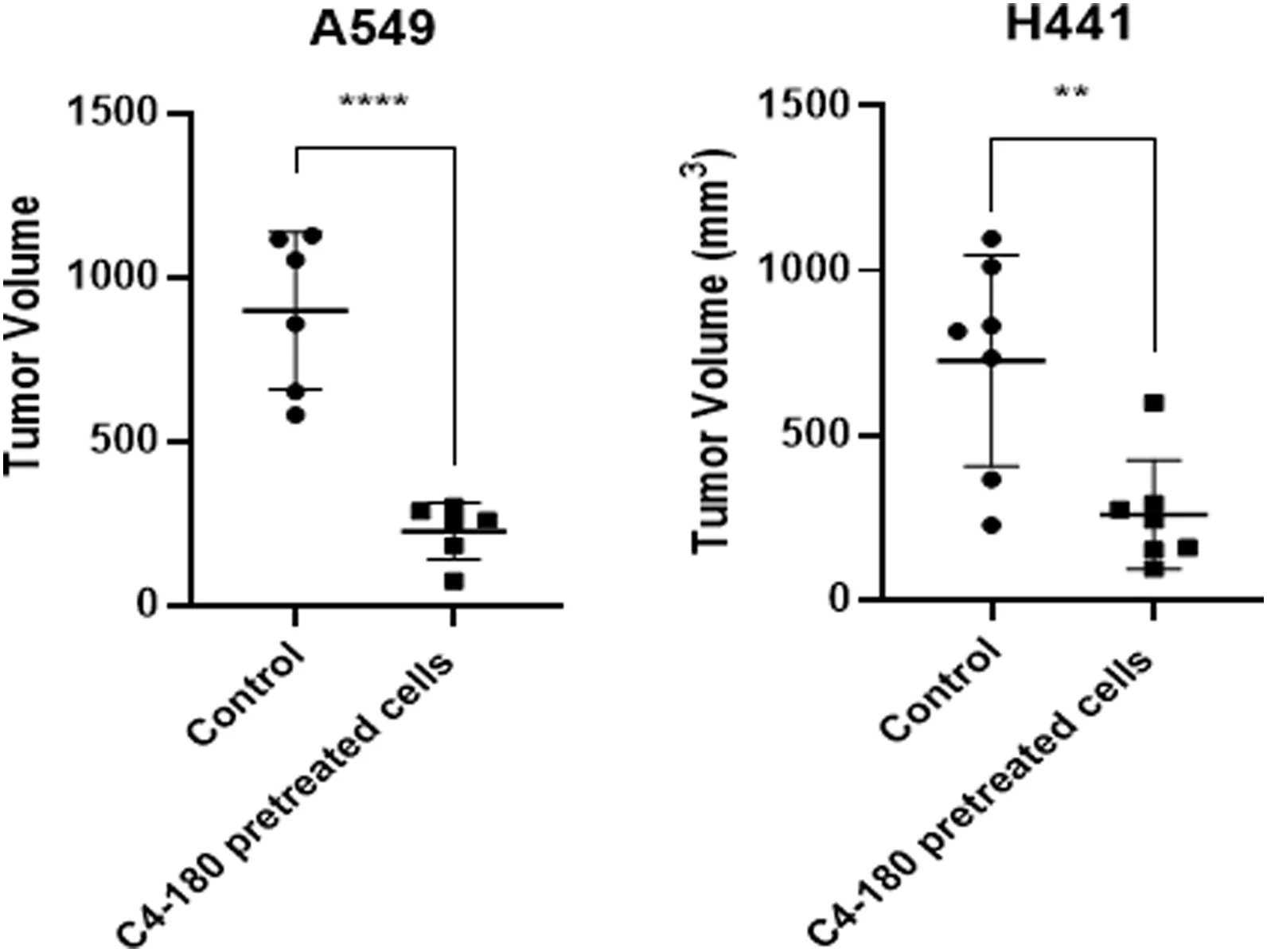
**Pretreatment of lung cancer cells with C4-180 suppresses formation of tumors *in vivo*.** (*Upper Panel*) A549 and H441 cells were pre-treated *in vitro* with either DMSO or C4-180 (10 μM), followed by subcutaneous grafting of 1 × 10^6^ viable cells per mouse into the lower. right flank of NSG mice. Tumors were allowed to grow for a period of 4 weeks without further treatment, after which tumor volumes were measured by caliper to determine effects in treated vs control cells. Each point represents a single tumor in a single mouse. *t* test was used to determine significance (∗∗ = *p* < 0.01). Error bars are SD.

### C4-180 suppresses tumorigenesis in lung tumor models

Based on its anti-CSC properties, we anticipated that the drug would have anti-tumor effects in NSCLC. The *in vivo* activity of C4-180 was investigated using a human NSCLC cell line xenograft and KRAS LSL G12D transgenic model. 1 × 106 A549 cells (KRASG12S) were grafted into NSG mice and allowed to reach a tumor volume of 100 mm^3^ before being randomly assigned to either control or treatment groups (40 mg/kg C4-180 by IP daily or 0.75 mg/kg doxorubicin by IV once weekly served as standard of care comparator). Treatment was carried out over a period of 2 weeks, with weights taken weekly and measurements by caliper every other day. C4-180 significantly suppressed tumor growth when compared to control, comparable to treatment with doxorubicin (Fig. 6*A*). No significant effects on weight, BUN, or ALT levels were observed, suggesting there is little short-term toxicity from the agent.

**Figure 6:**
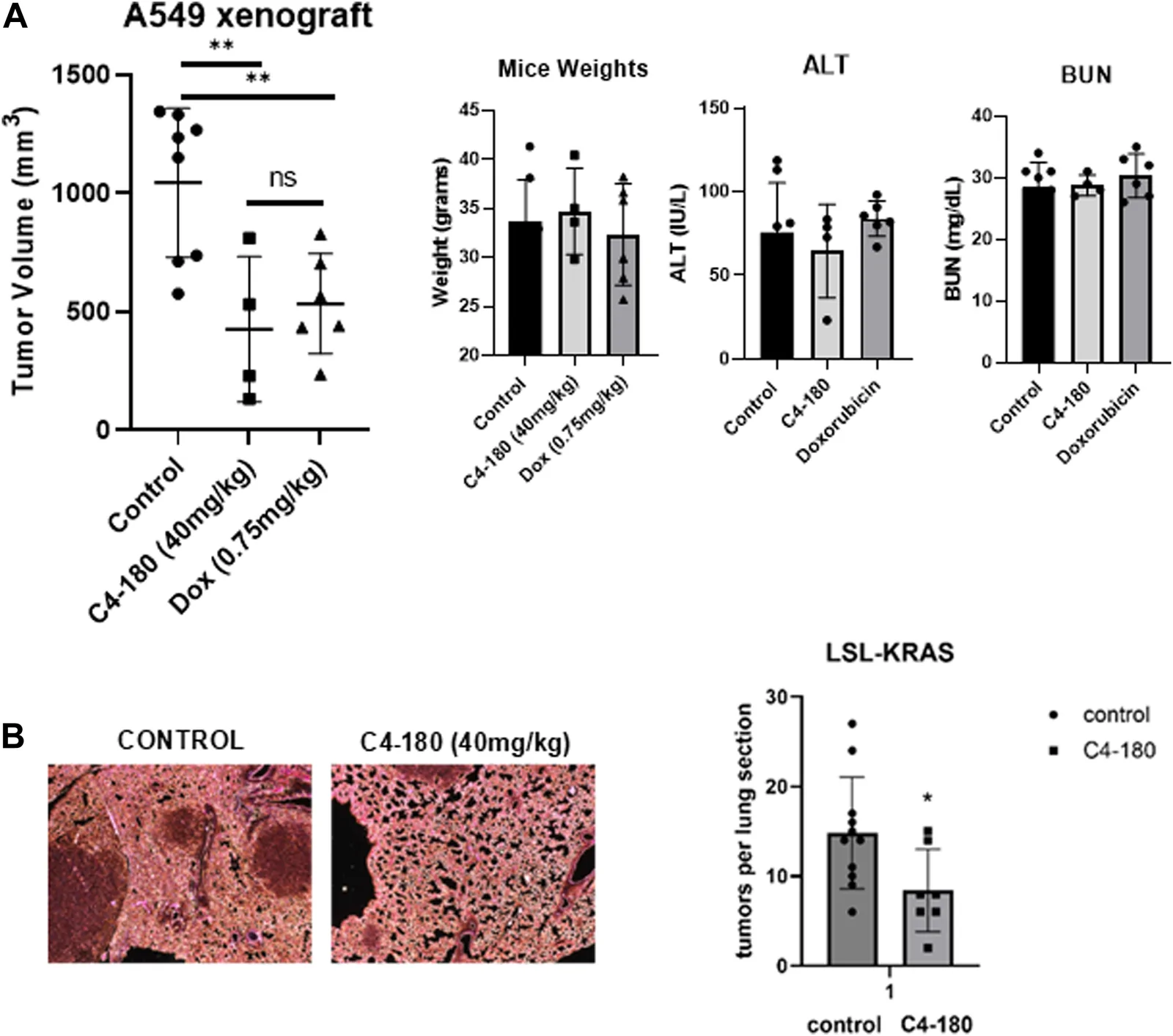
**C4-180 demonstrates activity against lung cancer models *in vivo*.***A*, 1 × 10^6^ A549 cells per mouse were grafted into the *lower right* flank of NSG immunodeficient mice by subcutaneous injection. Tumors were allowed to reach a volume between 100 - 150 mm^3^, after which mice were randomly placed into three different experimental groups and dosed accordingly: Control (DMSO, daily, intraperitoneal administration), C4-180 (40 mg/kg, daily, intraperitoneal administration), or doxorubicin (0.5 mg/kg, once weekly, intravenous tail injection). Tumor volume measurements were taken 3 times a week. Weights were taken once a week to ensure any weight loss remained under 10%. After a 2-week treatment period, mice were sacrificed to determine the effect on drug treatment on tumor growth. ALT and BUN assays were conducted on blood samples taken retro-orbitally prior to sacrifice at the end of the experiment. (*t* test used for significance; ∗∗ = *p* < 0.01). Error bars are SD. *B*, effects of C4-180 on tumor formation in the KRAS LSL-G12D transgenic model: Ad5mSPC-Cre (University of Iowa Viral Vector Core) was diluted to 2 × 10^6^ plaque forming units (PFU) per 20uL in Minimum Essential Media supplemented with calcium chloride, followed by administration of 20 uL of Ad-Cre/MEM mix to the lungs through oropharyngeal aspiration. Mice were randomized into control and C4-180 (40 mg/kg) treatment groups 2 weeks after Cre was administered, then dosed daily for a 2-week period by intraperitoneal injection. Following the 2-weeks treatment period, mice were sacrificed, lungs removed, sectioned and stained with H&E (*left panel*) by the University of Louisville Pathology Core. The total tumors per lung section were then scored (*right panel*). (*t* test used for significance; ∗ = *p* = 0.0336). Error bars are SD.

The KRAS LSL-G12D transgenic model is a well-characterized STOP-lox system that allows mutant KRAS to be activated upon cre-administration, leading to the spontaneous formation of RAS-driven adenocarcinoma endogenously within the mouse lung (43). Gene activation was induced by oropharyngeal administration of Ad-Cre (44). 2 weeks after cre-treatment, animals were dosed with C4-180 once per day for 2 weeks. Animals were then sacrificed and lungs isolated for histopathology analysis. Scoring of two slides per lung for each animal showed a significantly lower level of tumor development in the C4-180-treated animals (Fig. 6*B*).

## Discussion

RAS is the most frequently mutated oncogenic driver of NSCLC tumors (3, 5). Moreover, other oncogenic mutations commonly linked to lung cancer, such as those in EGFR and HER2, act upstream of RAS (3, 4), so even in wild type RAS tumors, the RAS protein may be hyper-activated. For these reasons, RAS has long been considered the most attractive therapeutic target in NSCLC.

RAS is thought to use three main mitogenic signaling pathways to modulate cellular homeostasis (6). The RAF/MAPK pathway and the PI-3K/AKT pathway are the best studied, shown to regulate key processes involved in cellular proliferation and survival (45, 46). The least studied pathway is the RAS/RALGEF/RAL pathway, where RAS binds and activates exchange factors for the RAS-related small G-proteins RALA and RALB. This results in promoting the formation of active, GTP-bound RAL proteins.

Although the RAS/RALGEF pathway only exerts modest effects on 2D growth in *vitro* compared to Raf and PI3-kinase (47, 48), it appears critical for 3D growth, tumorigenesis, and metastasis (11, 16, 19, 20, 21, 22, 23). Activation of the RAS/RALGEF pathway is necessary for RAS-driven tumor growth *in vivo* (19), whereas knockout of RALGDS suppresses the ability for RAS-driven tumor formation (20). RAL pathway activation drives the ability of non-metastatic prostate cancer cells to metastasize to bone (21), while RAL proteins are necessary for pancreatic cancer cell metastasis to the liver (49). Furthermore, RALGDS-transformed 3T3 fibroblasts demonstrate a greater metastatic spread when compared to those transformed by a constitutively active Raf mutant (50). The apparent lack of importance to 2D growth and survival but the essential role in tumorigenesis/metastasis may make this pathway a highly valuable therapeutic target in cancer with limited toxic potential. To date no approved inhibitors of this pathway have been developed.

Up until 2021, due to the difficulty in targeting RAS itself, efforts had focused on targeting the downstream signaling pathways of RAS, and numerous Inhibitors of the MAPK and PI3K pathways have received FDA approval (51). However, these agents have proved of limited utility as single agents and have a low response rate when used in combination (52). The situation changed with the approval of the KRAS^G12C^-targeted drugs Sotorasib and Adagrasib for refractory NSCLC-treated tumors. These agents both take advantage of the cysteine residue at the mutant site to form a covalent bond with the RAS protein, resulting in its inactivation (53).

However, although these drugs broke through the “undruggable” reputation of RAS, they have proved disappointing in the clinic. Relatively rapid resistance develops, resulting in an overall survival rate only modestly better than previous standard of care (26). The molecular mechanisms underlying the resistance are currently an area of intense scientific investigation. However, the underlying reason that allows resistance to develop may be the failure of the compounds to act as expected and suppress all the RAS signaling pathways.

Although Sotorasib is highly potent at blocking the RAS/MAPK pathway, treatment of cells can stimulate activation of PI3K/AKT pathway and has little effect on the RAL pathway (27, 28, 54, 55). As these additional pathways have been strongly implicated in cell survival, and the maintenance of cancer stem cells (11, 12, 13, 46), it is perhaps not surprising that Sotorasib has limited long term efficacy in the clinic. The reasons why these direct RAS inhibitors do not suppress all the RAS pathways are also not fully understood. However, the presence of wild type RAS proteins, RAS related proteins and activation feedback loops may play important roles (56, 57, 58).

Thus, although direct RAS inhibitors have now been developed for lung cancer, they do not appear to be as effective against all RAS signaling pathways as one might have anticipated. This may limit their therapeutic value as single agents in lung cancer. We have previously described the first pan-RALGEF inhibitor (C4-180) and its activity against pancreatic model systems (28). Here we sought to determine if it is also active in NSCLC systems and to identify the mechanisms by which it acts.

We have found that the agent is active *in vitro* and *in vivo* against multiple models of RAS driven NSCLC. Our observations *in vitro* confirm that the agent is effective against 3D growth models, in line with observations from genetic inactivation studies (19, 20, 30, 47). We then examined the potential mechanisms of action of C4-180.

The best-characterized terminal effector of the RAS/RALGEF/RAL pathway is the protein RALBP1, most commonly implicated in its role in regulating endocytosis. However, it also regulates the transport of electrophiles out of the cell to regulate cellular detoxification (59). Cancer cells utilize this function as a mechanism of resistance, with RALBP1 mediating the efflux of a range of chemotherapeutics, the most characterized being doxorubicin (60, 61, 62). To determine if we were suppressing RALBP1 function by inhibiting RAL, we examined the effects of our inhibitor on doxorubicin transport and toxicity. In line with previous evidence demonstrating the effects of RALBP1 knockdown and doxorubicin treatment (60, 61), we observed an increased accumulation of doxorubicin in cells pre-treated with C4-180, coinciding with a significant increase in cell death compared to doxorubicin treatment alone (Fig. 2). Although an effective chemotherapeutic, doxorubicin is limited by cardiotoxicity. It is possible that by blocking the function of RALBP1, which is often overexpressed in tumors, it might be possible to use reduced doses of doxorubicin to limit toxicity whilst retaining anti-tumor efficacy. We did perform combination studies in mice, but we lost most of the cohort due to reaching humane endpoints early. This may support the concept *in vivo*, but it would require larger scale dosing studies to determine if there is any potential for tumor specificity. This is a future ambition.

Another major effector system modulated by RAL proteins is the exocyst (63). The exocyst is an eight sub-unit complex that normally regulates vesicular trafficking and polarized exocytosis. Furthermore, the exocyst associates with RAB-positive recycling endosomes to facilitate their transport to the membrane (36), and the Wnt receptor Fzd has been shown to be directed to recycling endosomes (37). Thus, as an indication of whether we may be modulating exocyst dynamics, we utilized a GFP-tagged Fzd receptor and measured its sub-cellular localization after treatment by fluorescent microscopy. We observed a blocking of the cycling of Wnt receptor Fzd4 normally expressed on the cell membrane, with C4-180 treatment causing a greater colocalization with perinuclear-localized lysosomes (Fig. 3), suggesting impairment of exocyst cycling. There are several other, less well characterized RAL effector systems, and we would predict that they too will be inhibited.

RAL GTPases are highly activated in the CSC subpopulation and necessary for stem cell function (11, 16). CSCs are a unique subpopulation of intratumoral cells that are capable of self-renewal and differentiation. They have been implicated in tumor relapse, metastasis, and therapeutic resistance due to their ability to give rise to new tumors *in vivo*, as well as the expression of transporters that mediate efflux of chemotherapeutic agents (17, 18, 38). Knockout of RAL GTPases has been shown to abrogate stem cell function through a RALB/SEC5/TBK1 pathway (15). RAL can also regulate stem cell function through Wnt signaling (13). Moreover, RALBP1 has been implicated in CSC survival (39). Treatment with our agent was sufficient to suppress the expression of stemness markers *in vitro* of NSCLC cell lines. The cell lines used contained 12S and 12V mutations in K-RAS. Consequently, we used a pre-clinical pan-K-RAS inhibitor (BI-2865), not Sotorasib (which only works on KRASV12C), as a control. The C4-180 RALGEF inhibitor effectively suppressed the CSC compartment, as measured by CSC marker protein expression. However, the pan-K-RAS inhibitor had little effect on the RAL pathway or the CSC compartment in these mutant K-RAS cells, despite totally suppressing the MAPK pathway. As a gold-standard assay for the presence of CSC in the cell population, we pre-treated cells until suppression of CSC markers was observed, then used these pretreated cells to perform xenograft transplantation. In these experiments, no further treatment was used after injecting the same number of live cells. The pre-treated cell populations, depleted of CSC, failed to generate tumors.

These findings suggest that the RAL pathway is not actually under the control of the mutant K-RAS in these cells. As the C4-180 agent works by binding to the RA domain of RALGEFs and preventing their interaction with RAS (28) (thus suppressing RAL activation), its efficacy suggests that it is blocking something from binding to the RA domain of RALGEFs, just not the mutant K-RAS. The anti-K-RAS agents BI-2865 or Sotorasib are not effective on H- or N-RAS. In addition, there are multiple RAS-related proteins that can bind RALGEFs, such as Rit and R-RAS (64, 65). These may be the source of the K-RAS-independent activity. These findings also suggest that RAS inhibitors that do not suppress RAL and the CSC compartment are likely to perform poorly long-term in the clinic as single agents.

We have previously observed anti-tumor effects of C4-180 against pancreatic tumor systems containing activated RAS (28). Here we observe similar effects in NSCLC cell lines. However, subcutaneous cell line xenografts in immunocompromised mice have limited physiological relevance to a primary tumor growing in the lung. To address this limitation, we also examined the effects of the drug on a transgenic model of lung tumor induction. In this system, expression of KRAS^G12D^ mutant protein upon cre administration leads to the spontaneous formation of adenocarcinomas endogenously within the mouse lung. Similar to our cell line xenografts, we also observed suppression of tumor development. In each case, we observed no obvious adverse effects on the animals and analysis of liver and kidney function showed that they remained within normal parameters.

Thus, we demonstrate a novel pan-RALGEF inhibitor that has anti-tumor and anti-CSC activity in NSCLC with no obvious toxicity. We hypothesize the use of such an agent in combination with anti-RAS small molecule inhibitors could allow full RAS signaling cassette shutdown and lead to more permanent tumor regressions.

## Experimental procedures

### Tissue culture, cell lines, antibodies, reagents

A549 and NCI-H441 cells were purchased from ATCC and cultured according to the supplier’s recommendations. Cells were batch frozen after first passage and fresh aliquots thawed out after every ∼ 4 months. RALA antibody (lot: 3930954) was provided with RAL activation assay kit (Millipore Sigma, Lot: 3683231). CD44 (Cat.# 3570S), P-ERK (Cat.# 9101L), and ERK (Cat.# 9102L) antibodies were from Cell Signaling Technology. ALDH1A3 antibody (ab129815) was purchased from Abcam. MEK inhibitor trametinib was purchased from Selleckchem (Cat.#S2673). KRAS inhibitor BI-2865 (Cat.# HY-153724) and KRAS^G12C^ inhibitor Sotorasib (Cat.# HY-114277) were both purchased from MedChemExpress.

### 3D soft agar colony formation assays

Were performed essentially as described previously (28). IC50s for each cell line were determined using GraphPad Prism.

### 2D treatment and RAL activation assays

Cells were treated with either DMSO, C4-180 (10 μM), BI-2865 (2.5 μM) for 2 h (under which conditions the RAS inhibitors completely suppress phospho ERK) before effects on RAL pathway activation were determined using a commercial RAL Activation Assay Kit (MilliporeSigma) according to the manufacturer’s instructions. For determination of effects on CSC markers by Western blot, CD44, and ALDH1A3 antibodies were used.

### Doxorubicin transport and combination treatment assay

Cells were pre-treated with either DMSO or C4-180 (10 μM) for 2 h, followed by an hour of treatment with 10 μM doxorubicin (Selleckchem, catalog no. S1208). Once cells were refreshed with drug-free media, red fluorescence was detected and imaged using the EVOS M5000 microscope at 1 h and after 24 h. Total red fluorescence area for each time point and condition was measured using ImageJ software. For drug combination assays, cells were seeded in duplicate and treated with either DMSO, doxorubicin, C4-180, or a combination of C4-180 and doxorubicin. After incubation for 3 days, cells were imaged and viable cells counted by hemocytometer.

### Aldefluor ALDH activity assay

ALDH activity was determined using a commercial ALDEFLUOR kit (STEMCELL Technologies, lot no. 1000112877) according to manufacturer’s protocol. Briefly, after a 1-week treatment period with either DMSO, C4-180 (2.5 μM), or BI-2865 (2.5 μM), cells were trypsinized and resuspended in the provided assay buffer at a concentration of 1 × 10^6^ cells/ml. Four different conditions were assessed: DEAB negative control (specific inhibitor of ALDH isoforms), DMSO-positive control, and C4-180 or BI-2865 treated cells. After addition of 5 μl DEAB to its appropriate tube, 2.5 μl of Aldefluor substrate was added to each condition, mixed well, and allowed to incubate for 1 h in a 37 °C water bath. Tubes were mixed gently every 15 min to avoid settling off cells. Following incubation, cells were placed on ice and analyzed using flow cytometry to determine percentage of side scatter low, ALDH^high^ cells for each condition based on gating established by DEAB control.

### Frizzled/lysosome immunofluorescence assay

A549 cells were transfected with 2 μg of Fz4-GFP plasmid (Addgene, Plasmid #42197) using jetPRIME© transfection reagent. After 24 h, cells were treated with either DMSO, 100 nM Trametinib (Selleckchem, catalog no. S2673), or C4-180 (10 μM) and allowed to incubate for another 24 h. Treated cells were then stained with 75 nM LysoTracker Deep Red (Invitrogen, catalog no. L12492) for 30 min and imaged using an EVOS M5000 microscope. Colocalization with lysosomes was confirmed using the JaCoP plugin for ImageJ. Percentage of membrane vs. perinuclear localized Fz4-GFP was determined for each condition by manually scoring a total of 100 cells.

### Cell line xenografts

All animal experiments were pre-approved by the IACUC committee of the University of Louisville. Experiments were performed with an approximately equal mix of male and female animals. 1 × 106 cells were grafted by subcutaneous injection into the lower right flank of NSG (NOD-*scid* IL2Rgamma^null^) immunodeficient mice. Tumors were allowed to reach a volume of between 100-150 mm^3^, after which they were assigned to either experimental group (DMSO or C4-180). Mice were then dosed daily by intraperitoneal injection at 40 mg/kg for a period of 2 weeks. Tumor volume measurements were taken every other day, and weight measurements once a week, for the duration of the treatment period. For pre-treatment assays to determine effect on tumor formation, A549 and H441 cells were treated at the same concentration and time point which CSC marker suppression was observed, before viable cells were grafted into NSG mice. Once palpable, tumors were measured once weekly.

### MTT Assay

As a measure of the percentage of viable cells grafted into the mice, an MTT Assay Kit (abcam, Cat.# ab211091) was used according to manufacturer’s protocol. Percent viability of cells in comparison to DMSO vehicle control was calculated using the formula: *%Viability = [(100 × (Control – Sample))]/Control]*.

### Transgenic LSL KRAS G12D mice

All animal experiments were pre-approved by the IACUC committee of the University of Louisville. Experiments were performed with an approximately equal mix of male and female animals. KRAS^LSL-G12D^ mice, which contain an endogenous KRAS G12D mutation preceded by a stop cassette flanked with a Lox-Stop-Lox sequence, were purchased from The Jackson Laboratory. As previously described, excision of the stop cassette through administration of Cre recombinase virus leads to the expression of the KRAS^G12D^ mutant, promoting the spontaneous development of lung adenocarcinoma (66). Tail snip was used for genotyping, which was confirmed by PCR by *Mouse Genotype* (Escondido, CA). Ad5mSPC-Cre (University of Iowa Viral Vector Core, lot: Ad4703) was diluted to 2 × 10^6^ plaque forming units (PFU)/20 μl of MEM supplemented with 10 mM calcium chloride, allowing for a final volume of 20 μl to be administered per mouse. Cre was introduced through oropharyngeal administration (44). Mice were randomized into control and C4-180 (40 mg/kg) treatment groups 2 weeks after Cre administration, followed by treatment daily for 2 weeks after which mice were sacrificed. Lungs from each treatment group were dissected, allowing for quantification of tumors per lung section following H&E staining.

## Data availability

The datasets generated during and/or analysed during the current study are available in the Figshare repository: https://figshare.com/account/items/30896351/edit.

## Conflict of interest

The authors declare the following financial interests/personal relationships which may be considered as potential competing interests: The work was funded by a generous grant from the American Lung Association to GJC. GJC holds a patent on the inhibitor compounds.
